# The importance of termites and fire to dead wood consumption in the longleaf pine ecosystem

**DOI:** 10.1038/s41598-021-03621-0

**Published:** 2021-12-16

**Authors:** Michael D. Ulyshen, Thomas N. Sheehan

**Affiliations:** 1 USDA Forest Service, 320 Green Street, Athens, GA 30602 USA; 2 The Jones Center at Ichauway, 3988 Jones Center Drive, Newton, GA 39870 USA

**Keywords:** Fire ecology, Forest ecology, Entomology

## Abstract

Microbes, insects, and fire are the primary drivers of wood loss from most ecosystems, but interactions among these factors remain poorly understood. In this study, we tested the hypothesis that termites and fire have a synergistic effect on wood loss from the fire-adapted longleaf pine (*Pinus palustris* Mill.) ecosystem in the southeastern United States. We predicted that the extensive galleries created by termites would promote the ignition and consumption of logs by fire. We exposed logs from which termites had or had not been excluded to prescribed fire after 2.5 years in the field. We found little support for our hypothesis as there was no significant interactive effect of termites and fire on wood mass loss. Moreover, there was no significant difference in mass loss between burned and unburned logs. Termites were responsible for about 13.3% of observed mass loss in unprotected logs, a significant effect, while microbial activity accounted for most of the remaining mass loss. We conclude that fire has little effect on wood loss from the longleaf pine ecosystem and that termite activity does not strongly promote wood combustion. However, longer term research involving multiple burn cycles, later stages of decay, and differing fire intensities will be needed to fully address this question.

## Introduction

The breakdown of woody debris in forests is largely driven by three primary factors: microbes (including fungi), insects, and fire^1–4^. The relative importance of these determinants can vary greatly among ecosystems. While microbial activity drives wood decomposition in many boreal or temperate zones, for example, insects can exceed microbes in importance in the tropics where termites are active^5,6^. The importance of fire to wood consumption is also highly variable depending on differences in fire frequency and severity as well as local climate conditions. Although few studies have attempted to assess the relative contributions of microbes, insects, and fire to wood consumption, even less is known about how these factors may interact to speed up or slow down wood loss from ecosystems.

Interactions among microbes, insects, and fire are known to exist but remain poorly understood and may have inconsistent effects on wood consumption. Although wood-boring beetles are known to vector wood-rotting fungi^7^ and may facilitate fungal establishment by creating tunnels through bark and into wood^8^, for example, some insects are known to alter fungal community composition in ways that have been shown to reduce decay rates^9^. Fire is also known to change the trajectory of fungal communities in dead wood^10^ and can slow decay rates by reducing fungal hyphal lengths as well as fungal activity^11^. Previous studies exploring the impacts of fire on wood-dwelling insects suggest that termites are highly resistant to fire and that low-intensity burns have little effect on wood-dwelling beetle communities^12,13^. Although certain forest pests (e.g., bark beetles) are known to increase wildfire risk due to increased fuel loads^14^, little is known about how the activities of insects within dead wood influence fire behavior. Termites and other insects create extensive galleries within dead wood^15^ and this likely improves aeration with the possibility of facilitating wood combustion. Here we present the results from a study aimed at testing this idea in the fire-adapted longleaf pine ecosystem of the southeastern United States.

Originating approximately 7500–5000 years ago, the longleaf pine ecosystem was the dominant ecosystem on the southeastern U.S. coastal plain at the time of early European colonization^16^. It has since been largely displaced by other land uses and is now considered one of the most endangered ecosystems in North America, occupying just 3% of its historic range^16,17^. The longleaf pine ecosystem is characterized by widely spaced trees (mostly longleaf pine) in the overstory, supports high herbaceous plant diversity on the forest floor and provides habitat for thousands of arthropod species^18^. The open pine-dominated conditions are maintained by some of the shortest fire return intervals on the continent^19^, with fires historically occurring every 1–2 years as initiated by lightning strikes and later by Native Americans^16^. Prescribed fire remains one of the most important tools used by managers to preserve and restore this ecosystem.

The volume of coarse woody debris is lower in the longleaf pine ecosystem than in many other forest types^20^. While this is partly explained by the low basal area characteristic of this ecosystem, dead wood also appears to be consumed quickly through the actions of microbes, termites, and fire. However, this rapid consumption appears to be limited to the outer layers of sapwood whereas the inner core of heartwood that forms as longleaf pines age is much more persistent (Supplementary Fig. S1) and appears to accumulate over time^20^. The role of fire in reducing dead wood volumes in the longleaf pine ecosystem remains unclear. Although Hanula et al.^21^ found no significant differences in dead wood volumes among different burn frequency treatments in Florida, casual observations following a burn suggest that individual logs or standing dead trees can be mostly or even entirely consumed by fire. The effects appear to vary greatly among dead wood items, however, and it remains unclear why some logs combust while others experience only minimal scorching. Conditions within the logs probably play an important role. For example, it is well established that combustion is inhibited by high wood moisture^4^. In addition, high wood porosity promotes both ignition and sustained smoldering^4^.

Although the importance of insects to the decomposition of longleaf pine has not been investigated, previous work on *Pinus taeda* L., another species native to the southeastern U.S., suggests the five species of native subterranean termites (*Reticulitermes*) may consume as much as one fifth of dead wood volume within the region^15^. These insects excavate extensive galleries, giving the wood a spongy appearance (i.e., high porosity) when viewed in cross section (Supplementary Fig. S1A). Other wood-feeding and excavating insects like longhorned beetles (Coleoptera: Cerambycidae) and carpenter ants (Hymenoptera: Formicidae: *Camponotus* spp.) produce less extensive galleries but also contribute to the increasing porosity of dead wood as decomposition proceeds. Because the galleries of wood-feeding insects, especially termites, can be expected to facilitate airflow and were shown previously to reduce the water content of rotting logs^15^, we hypothesized that termites and fire have a synergistic effect on wood loss from the longleaf pine ecosystem.

## Methods

### Study location and design

This study was conducted at The Jones Center at Ichauway, a research center located on the Coastal Plain of southwestern Georgia, USA (31.27, −84.48). Ichauway has a subtropical climate with mean daily temperature range of 21–34 °C in the summer and 5–17 °C in the winter and annual rainfall of 131 cm^22^.

We felled two healthy longleaf pine trees of similar size growing ~ 20 m apart from which a total of 48 logs ~ 35 cm in length and ~ 17 cm in diameter (range:13.9–21.4) were cut. Because removing bark and drying the logs would have disrupted the natural colonization patterns of insects and fungi, we estimated their initial dry wood weights based on data collected from ~ 3 cm disks cut adjacent to both ends of each log. We recorded the diameter, length, and wet weight of each log and disk. We then removed the bark and phloem from the disks and dried them at 70 °C until no further mass loss was observed. We divided the final dry wood weight of each disk (without bark) by the initial wet weight of each disk (with bark) to determine what proportion of each disk consisted of dry wood. We averaged these values for the two disks adjacent to each log and multiplied this product by the initial weight of the intervening log to estimate the initial dry wood weight of each log. We also measured the diameter of heartwood visible on the disks to include percent heartwood as a covariate in the model. The logs were measured and stored in a climate-controlled building until we placed them in the field on 10 September 2018, four days after they were cut.

**Figure 1:**
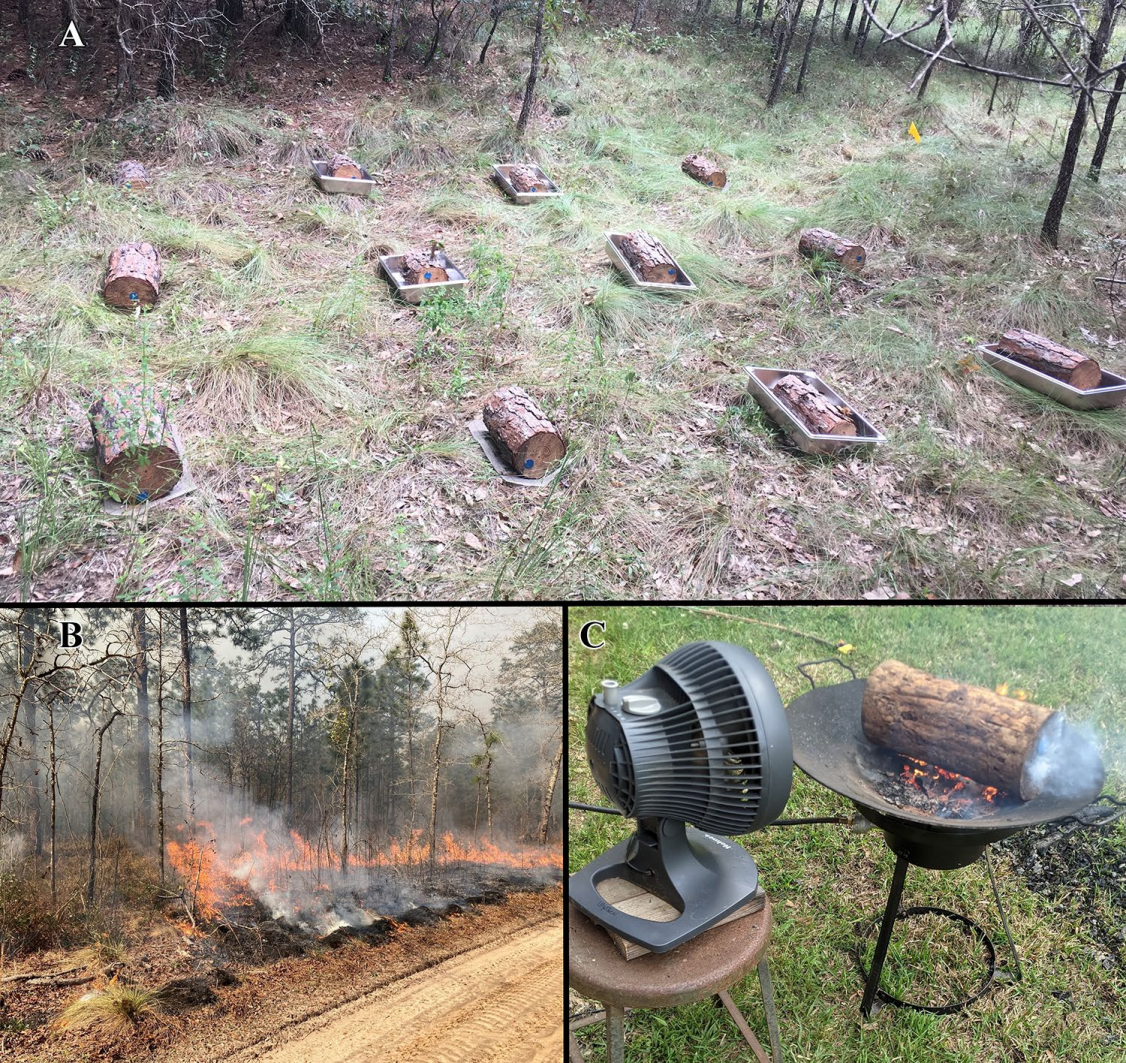
Logs assigned to the different termite treatments at one of the locations are visible in image **(A)**. Image **(B)** shows one of the prescribed fires and image **(C)** shows the method for extracting soil imported into the logs by termites.

Each log was randomly assigned to one of four termite treatments (Fig. 1A). One third of them (n = 16) were assigned to the “closed pan” treatment which excluded termites. Each closed pan consisted of a stainless-steel food tray (Vollrath Super Pan V item number 30040: 52.6 cm × 32.0 cm × 9.4 cm) with a large rectangular hole (40.6 cm × 20.3 cm) cut out of the bottom. A sheet of fine stainless-steel mesh screen with 0.3 mm openings was bolted in place over the hole to permit colonization of the wood by fungi from the soil. Pans of nearly the same design (only slightly shallower) were shown to be effective at excluding termites in a previous study^23^. The remaining logs (n = 32) were assigned to one of three treatments unprotected from termites. Eight of them were assigned to the “open pan” treatment which consisted of pans identical to the closed pans except for three rectangular openings (2.5 × 19 cm and separated by 7.5 cm) cut through the screen mesh to allow termite colonization. Twelve others were assigned to an “open screen” treatment which consisted of loose screens (i.e., not in pans) identical to those used in the open pan treatment. We did not expect to see any differences between open pan and open screen treatments in terms of termite colonization or decomposition and only included this second treatment to compensate for a shortage of pans. The remaining twelve logs were assigned to a “bare ground” treatment which involved placing the logs in direct soil contact. This treatment was added to determine if the screens used in the other two unprotected treatments affected termite colonization or decay rate. We assigned twice as many logs to the unprotected treatments to ensure adequate termite colonization to test our primary research question. To reduce the risk to the study from unexpected disturbances, we divided the experiment among four locations separated by 25–50 m. The locations were all within a single stand that had not been burned since 2001^24^. Each location had two open pans, three open screens, three direct ground, and 3–5 closed pan treatments. We checked the logs every other month for any disturbances and were careful to remove any fallen branches from around the installations including anything that fell into the pans. Hurricane Michael caused widespread damage across Ichauway in October 2018 but did not affect this study.

We left the logs in the field for about two and a half years before exposing half of them to prescribed fire. On 5 March 2021, we examined each log externally for evidence of termite (*Reticulitermes* spp. (Blattodea: Rhinotermitidae)) and carpenter ant (*Camponotus* spp. (including *C. floridanus* (Buckley)) (Hymenoptera: Formicidae)) activity and also recorded a visual estimate of how much intact bark was remaining on each log. Because further bark loss could not be prevented while moving the logs, we decided to remove the bark from all of them for consistency. We randomly assigned half the logs from each treatment to the burn treatment while the other logs remained unburned. We moved the logs to be burned to a plot ~ 100 m away that was scheduled to be burned on the same day (Fig. 1B). Each log was placed on a clump of wiregrass (*Aristida stricta* Michx.) to ensure exposure to fire and the logs were separated by ~ 1 m. The fire burned at 108.6 °C on average, with an average maximum temperature of 392.7 °C, based on four data loggers placed 0.5 m above ground. Because the logs were quite damp, they did not burn well, with little charring visible. We therefore decided to expose them to a second fire scheduled for the following day. They were moved to a site ~ 1 km away that was burned on 6 March at 131.2 °C on average, with an average maximum temperature of 455.1 °C, based on five data loggers placed 0.5 m above ground. This second burn was hot enough to cause charring on many of the logs and combustion clearly occurred in some logs. We placed all logs, except for one that was still smoldering, in individual trash bags four hours after the burn. The remains of the last log were collected the following morning. To replicate handling loss that may have occurred when the logs assigned to the burned treatments were moved, we handled the logs assigned to the unburned treatment similarly, picking them up and setting them down within an area not scheduled to be burned.

All logs were then dried at 102 ºC until no further mass loss was observed. We then scraped off any soil and other non-woody debris from the outside of each log before weighing. The logs were then split lengthwise with a chisel and hammer to check for any internal evidence of termite or carpenter ant activity. Termite activity was recognized by the characteristic galleries they leave behind. Carpenter ant activity was confirmed by the presence of adult ants within the wood. Because termites are known to transport soil into the wood, we burned all logs with evidence of termite galleries to extract this soil following Ulyshen and Wagner^25^ (Fig. 1C). This soil weight was then subtracted from the final log weight to get the final wood weight remaining in each log. Although termites may also import organic material along with soil and this may result in an overestimation of final wood weight (and thus an underestimation of the effects of termites on mass loss), we suspect this had negligible effects on our results and are not aware of any methods for isolating externally-derived organic matter from decomposing logs.

Finally, we confirm that permission to perform this study, including felling longleaf pine trees and destructively sampling experimental logs (as described above), on the Jones Center property was granted by the Center’s director and we followed all local and national guidelines for conducting this research. No humans or vertebrates were involved in this study although some invertebrates inhabiting the logs were killed during the final drying process.

### Analysis

The response variable in this study was wood mass loss which was calculated as 1-w_f_/w_i_ where w_f_ is the final dry weight of each log (after subtracting the weight of any soil) and w_i_ is the estimated initial dry weight of each log (see above). All analyses were performed in R 3.6.1^26^. Mass loss was the response variable in linear mixed effects models using the lmer function within the lme4 package^27^. We began with a full model consisting of termite treatment, fire treatment, the interaction between termite and fire treatments, *Camponotus* (presence/absence), initial log diameter, percent heartwood and percent bark cover as fixed effects and with location as a random effect. One log, which was almost completely consumed by fire, was identified as an extreme outlier based on plots of residuals and Cook’s distance and was excluded from further analysis in order to achieve normality of residuals. We then re-ran the full model and produced pairwise comparisons of termite treatments using the packages emmeans^28^ and multcompView^29^ to determine if there were any significant differences among the treatments unprotected from termites. Because there were no such differences, we simply used the presence/absence of termites in all subsequent analyses. Starting from the full model described above, we used backward selection to drop non-significant terms one at a time including the termite x fire interaction term. We retained the fire term in the model even when found to be non-significant as the effect of fire on wood decomposition was one of our primary research questions. We used the r.squaredGLMM function in the MuMIn package to obtain marginal and conditional R-squared values for fixed effects and fixed plus random effects, respectively. The results are presented as estimated marginal means (EMM) with significance based on the Sidak adjustment.

## Results

### Patterns of insect colonization

The closed pan treatment successfully excluded termites with no logs assigned to that treatment being colonized by the end of the study. By contrast, termites colonized nearly all logs assigned to the unprotected treatments, with only one log from the bare ground treatment remaining uncolonized by the end of the study. Carpenter ants colonized 20.8% of logs including two that were assigned to the closed pan treatment.

### Main analysis

**Figure 2:**
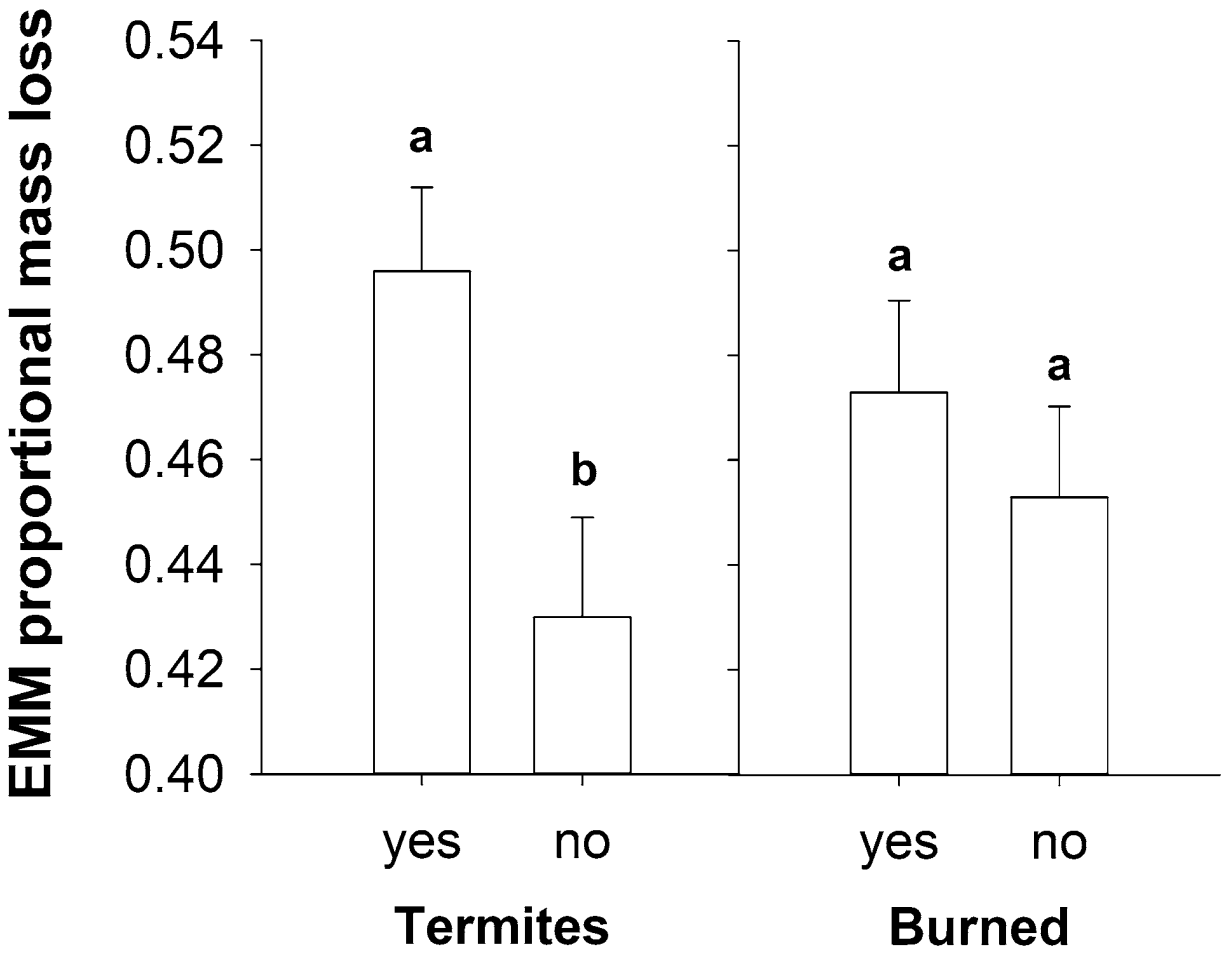
Estimated marginal means (EMM) ± SE proportional mass loss for the termite and fire treatments. Within each graph, different letters indicate a significant difference. These data come from a total of 30 and 17 logs with and without termites, respectively, and from a total of 23 and 24 burned and unburned logs, respectively.

Backward model selection resulted in a final model consisting of termites, fire and log diameter as fixed effects and included location as a random effect. Based on marginal and conditional R-squared values, the fixed effects explained 22.3% of variation while the fixed and random effects explained 30.8%. Logs colonized by termites lost significantly more mass than those without termites (estimate = 0.065, t_40.36_ = 3.35, P < 0.01) (Fig. 2). If the difference between expected marginal means for logs with and without termites (0.496–0.430 = 0.066) is taken to represent the contribution termites made to mass loss from the unprotected logs, then termites were responsible for about 13.3% of mass loss (0.066/0.496) from logs colonized by these insects. Log diameter had a negative effect on mass loss (estimate = -0.012, t_43.00_ = -2.11, P = 0.04). Unburned logs experienced slightly less mass loss than burned logs on average, but this difference was not significant (estimate = -0.020, t_41.03_ = -1.06, P = 0.29) (Fig. 2).

## Discussion

This study represents the first effort, to our knowledge, to quantify the relative importance of microbes (including fungi), insects, and fire to the consumption of wood from an ecosystem. Our findings suggest microbes are the primary drivers of wood decomposition from the longleaf pine ecosystem, contributing up to 86.7% of mass loss while termites were responsible for the remaining 13.3%. Fire was the least important of the three factors, with no significant difference in mass loss between burned and unburned logs. The fact that this was the case even after exposing the logs to two fires in quick succession lends support to the conclusion that fire has little short-term effect on dead wood pools in the longleaf pine ecosystem. Although this is consistent with the conclusion reached by Hanula et al.^21^, it is clear from casual observations that some logs or standing dead trees can be largely consumed by fire (MDU pers. obs.). We were able to capture this apparently rare phenomenon in the current study as evidenced by the one log that reached 97.2% mass loss after combusting during the second fire. It remains unclear why a few logs combust while most experience minimal charring (Supplementary Fig. S1C). This could relate to variability in fire intensity within a burn as well as conditions within the wood itself including resin content, wood moisture, and stage of decay^4^.

The primary purpose of this study was to test the hypothesis that termites and fire have a synergistic effect on wood loss. Although the interaction term was non-significant and therefore dropped during our model selection procedure, we cannot fully rule out the idea that termites may facilitate wood combustion. It is worth noting, for example, that burned logs with termite activity experienced slightly higher mass loss than the other treatment combinations (Supplementary Fig. S2). It is possible that we would have detected a significant difference with greater replication. It is also noteworthy that the one log that was almost completely consumed by fire in this study, which was dropped as an outlier from the analysis, had been colonized by termites. While we cannot reach any conclusions from this single observation, we also should not be too hasty in concluding that there is no meaningful interaction between termites and fire. Such a synergism could be both rare and ecologically meaningful if termites tip the balance in favor of near-complete combustion as opposed to external charring. Another important consideration is the potential effect of repeated fires over long periods of time. Stronger synergistic effects may be observed at later stages of decay than observed in this study given that wood ignites and smolders more readily as decomposition proceeds^4^. Another possibility is that termites compensate for reductions in fungal activity following fire^11^ given that they are largely unaffected by burns^12,21^. Research including multiple burn cycles would help clarify these relationships.

While this study is not the first to explore the effects of insects on wood decomposition, it is one of the first to do so without the confounding effects of mesh bags or insecticides used in most previous studies. By using open pans with screened bottoms, we avoided the potential for altering the microclimate surrounding protected logs relative to that of unprotected logs. Thus, unlike so many previous studies, we can be quite confident in our conclusion that termites were responsible for about 13.3% of mass loss observed at the end of the study. This finding helps resolve a question raised by Ulyshen et al.^15^. In that study, mesh cages and insecticides applied to the soil were used to exclude termites and other insects. Even though termites consumed about one fifth of wood volume, no effects on mass loss were observed. The authors suggested this may have been due to the stimulatory effect the insecticides may have had on decomposition, increasing fungal activity in the logs from which termites were excluded. Our results support this conclusion and are more in line with a mesh bag study by Ulyshen^30^ in which termites were responsible for about 13.7% of wood loss from pine logs after 31 months in upland sites in Mississippi. Based on a comparison of decay rates between caged and uncaged blocks, the author argued that the mesh enclosures were unlikely to have affected mass loss beyond their intended effect of excluding termites. Although the results from the current study support this conclusion, given the very similar results, using open pans rather than mesh bags to exclude termites in future investigations would help avoid such uncertainties, especially given the significant mesh effects reported in other studies^31,32^. However, the open pans have the disadvantage of not excluding other insects thus limiting their utility to termite studies. By not excluding wood-boring beetles and other insects from the protected logs in the current study, our results may underestimate the total importance of insects and overestimate the contributions of microbes to wood decomposition in the longleaf pine ecosystem.

Like other tree species possessing heartwood^33^, the decomposition of longleaf pine coarse woody debris is no doubt biphasic, requiring a multi-exponential function to account for differences in decay rates of sapwood and heartwood. The rapid degradation of the outer layers of sapwood is followed by the much slower deterioration of the heartwood center (Supplementary Fig. S1B), resulting in an accumulation of heartwood as stands age^20^. The current study focused on the first phase only, raising questions about the effects of microbes, termites, and fire over the second phase. Given the decay-resistant properties of heartwood, it is possible that termites and fire play more important roles in the second phase than they do in the first. Termites are commonly observed beneath pieces of heartwood and appear to feed on this material to some extent (Supplementary Fig. S1D). Carpenter ants, though found to be non-significant to decomposition in the current study, are also often active in heartwood remnants and could promote comminution (Supplementary Fig. S1E). Answers to these questions and a more thorough exploration of the interaction between termites and fire will require long-term study.

## Supplementary Information

Supplementary Figures.

## References

[1] Cornwell WK (2009). Plant traits and wood fates across the globe: Rotted, burned, or consumed?. Glob. Change Biol..

[2] Ulyshen MD (2016). Wood decomposition as influenced by invertebrates. Biol. Rev..

[3] Rayner ADM, Boddy L (1988). Fungal Decomposition of Wood: Its Biology and Ecology 587.

[4] Hyde JC, Smith AMS, Ottmar RD, Alvarado EC, Morgan P (2011). The combustion of sound and rotten coarse woody debris: A review. Int. J. Wildland Fire.

[5] Griffiths HM, Ashton LA, Evans TA, Parr CL, Eggleton P (2019). Termites can decompose more than half of deadwood in tropical rainforest. Curr. Biol..

[6] Wu C (2021). Stronger effects of termites than microbes on wood decomposition in a subtropical forest. For. Ecol. Manage..

[7] Jacobsen RM, Kauserud H, Sverdrup-Thygeson A, Bjorbækmo MM, Birkemoe T (2017). Wood-inhabiting insects can function as targeted vectors for decomposer fungi. Fungal Ecol..

[8] Leach JG, Orr LW, Christensen C (1937). Further studies on the interrelationship of insects and fungi in the deterioration of felled Norway pine logs. J. Agric. Res..

[9] Skelton J (2020). Fungal symbionts of bark and ambrosia beetles can suppress decomposition of pine sapwood by competing with wood-decay fungi. Fungal Ecol..

[10] Wikars L-O (2002). Dependence on fire in wood-living insects: An experiment with burned and unburned spruce and birch logs. J. Insect Conserv..

[11] Holden SR, Gutierrez A, Treseder KK (2013). Changes in soil fungal communities, extracellular enzyme activities, and litter decomposition across a fire chronosequence in Alaskan boreal forests. Ecosystems.

[12] Ulyshen MD, Lucky A, Work TT (2020). Effects of prescribed fire and social insects on saproxylic beetles in a subtropical forest. Sci. Rep..

[13] Ulyshen MD, Horn S, Barnes B, Gandhi KJK (2010). Impacts of prescribed fire on saproxylic beetles in loblolly pine logs. Insect Conserv. Divers..

[14] Billings RF (2004). Bark beetle outbreaks and fire: A devastating combination for Central America’s pine forests. Unasylva.

[15] Ulyshen, M. D., Wagner, T. L. & Mulrooney, J. E. Contrasting effects of insect exclusion on wood loss in a temperate forest. *Ecosphere* **5**, article 47 (2014).

[16] Van Lear DH, Carroll WD, Kapeluck PR, Johnson R (2005). History and restoration of the longleaf pine-grassland ecosystem: Implications for species at risk. For. Ecol. Manag..

[17] Noss, R. F. & Scott, J. M. *Endangered Ecosystems of the United States: A Preliminary Assessment of Loss and Degradation*. Vol. 28. (US Department of the Interior, National Biological Service, 1995).

[18] Folkerts GW, Deyrup MA, Sisson DC (1993). Arthropods associated with xeric longleaf pine habitats in the southeastern United States: A brief overview. Proc. Tall Timbers Fire Ecol. Conf..

[19] Guyette RP, Stambaugh MC, Dey DC, Muzika R-M (2012). Predicting fire frequency with chemistry and climate. Ecosystems.

[20] Ulyshen MD, Horn S, Pokswinski S, McHugh JV, Hiers JK (2018). A comparison of coarse woody debris volume and variety between old-growth and secondary longleaf pine forests in the southeastern United States. For. Ecol. Manag..

[21] Hanula JL, Ulyshen MD, Wade DD (2012). Impacts of prescribed fire frequency on coarse woody debris volume, decomposition and termite activity in the longleaf pine flatwoods of Florida. Forests.

[22] Goebel, P. C. *et al.**Forest Ecosystems of a Lower Gulf Coastal Plain Landscape: Multifactor Classification and Analysis*. 47–75. (2001).

[23] Ulyshen MD, Müller J, Seibold S (2016). Bark coverage and insects influence wood decomposition: Direct and indirect effects. Appl. Soil. Ecol..

[24] Kirkman LK (2016). Productivity and species richness in longleaf pine woodlands: Resource-disturbance influences across an edaphic gradient. Ecology.

[25] Ulyshen MD, Wagner TL (2013). Quantifying arthropod contributions to wood decay. Methods Ecol. Evol..

[26] R Core Team. *R: A Language and Environment for Statistical Computing (Version 3.6.1)*. http://www.R-project.org. (R Foundation for Statistical Computing, 2019).

[27] Bates D, Mächler M, Bolker B, Walker S (2015). Fitting linear mixed-effects models using lme4. J. Stat. Softw..

[28] Lenth R, Singmann H, Love J, Buerkner P, Herve M (2018). Emmeans: Estimated marginal means, aka least-squares means. R Package Version.

[29] Graves, S., Piepho, H.-P. & Selzer, L. multcompView: Visualizations of paired comparisons. *R Package Version* 0.1-7. (2015).

[30] Ulyshen, M. D. Interacting effects of insects and flooding on wood decomposition. *PLoS ONE* **9**, e101867 (2014). 10.1371/journal.pone.0101867 PMC4092066 25009985

[31] Stoklosa AM (2016). Effects of mesh bag enclosure and termites on fine woody debris decomposition in a subtropical forest. Basic Appl. Ecol..

[32] Kampichler C, Bruckner A (2009). The role of microarthropods in terrestrial decomposition: A meta-analysis of 40 years of litterbag studies. Biol. Rev..

[33] Mackensen J, Bauhus J, Webber E (2003). Decomposition rates of coarse woody debris—A review with particular emphasis on Australian tree species. Aust. J. Bot..

