# Supplementary material for: The importance of termites and fire to dead wood consumption in the longleaf pine ecosystem
Source: Sci Rep. 2021 Dec 16;11:24109. doi: 10.1038/s41598-021-03621-0 (PMC8677794; doi:10.1038/s41598-021-03621-0)

Supplementary Information

The importance of termites and fire to dead wood consumption in the longleaf pine ecosystem

Michael D. Ulyshen and Thomas N. Sheehan

Supplementary Figure S1. Termite galleries give wood a spongy appearance in cross section (A). In mature longleaf pine logs, the outer layers of sapwood degrade much more quickly than the heartwood core (B). Prescribed fire most commonly chars wood without significant combustion (C). Heartwood remnants with damage caused by termites (D, arrow) and *Camponotus* (E).

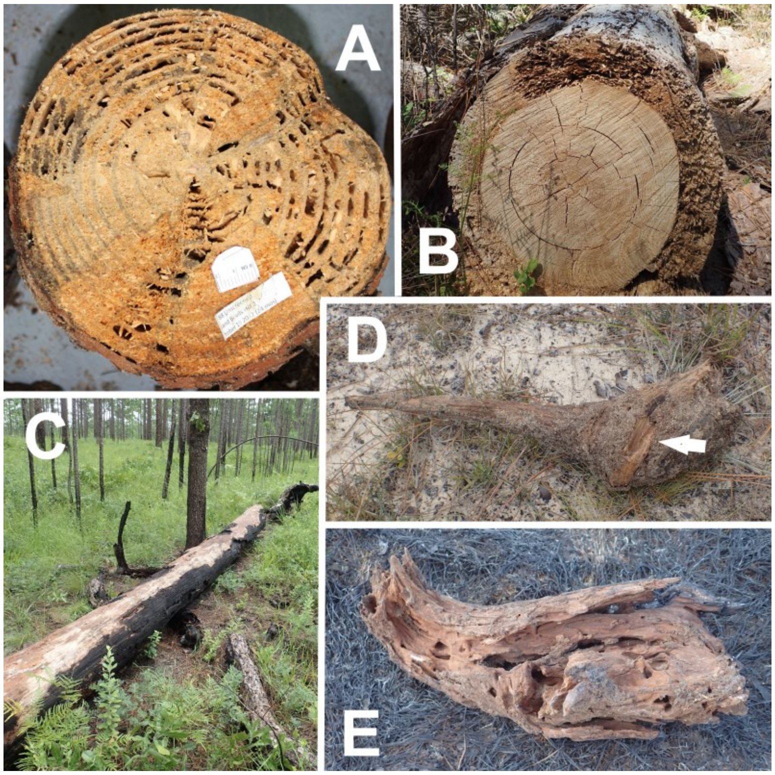

Supplementary Figure S2. Boxplots showing distribution of mass loss data for the different termite and fire treatment combinations. The boxes show the median value as well as lower and upper quartiles. The dots occurring beyond the whiskers are considered extreme values.

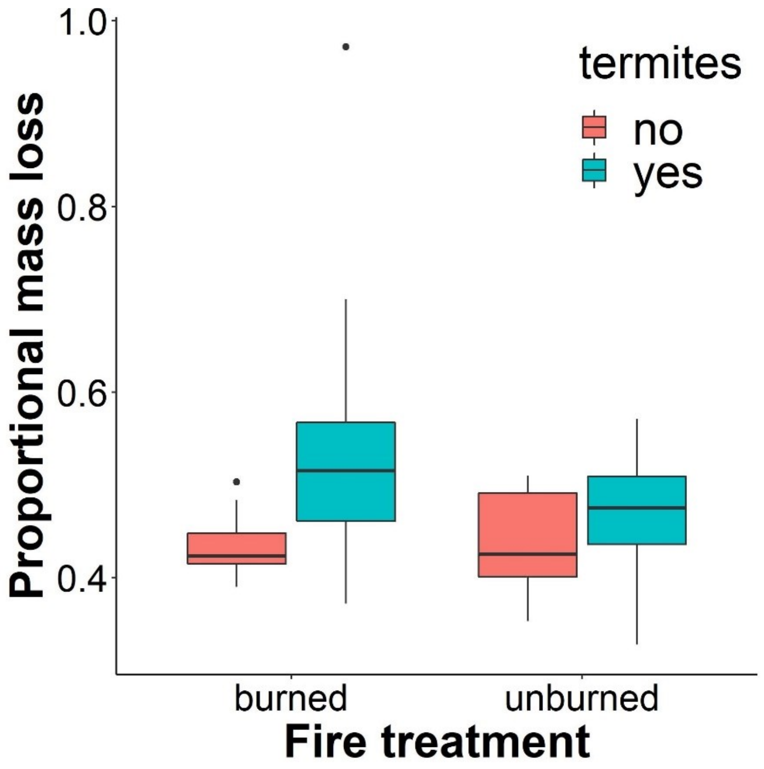

Supplement: Supplementary file 1 — Supplementary Figures. [file 41598_2021_3621_MOESM1_ESM.pdf]
